# Coping Skills Mobile App to Support the Emotional Well-Being of Young People During the COVID-19 Pandemic: Protocol for a Mixed Methods Study

**DOI:** 10.2196/23716

**Published:** 2020-10-08

**Authors:** Anna Serlachius, Kiralee Schache, Anna Boggiss, David Lim, Kate Wallace-Boyd, Jennifer Brenton-Peters, Elise Buttenshaw, Stephanie Chadd, Alana Cavadino, Nicholas Cao, Eva Morunga, Hiran Thabrew

**Affiliations:** 1 Department of Psychological Medicine University of Auckland Auckland New Zealand; 2 Psychological Medicine Counties Manukau Health Auckland New Zealand; 3 Epidemiology and Biostatistics School of Population Health University of Auckland Auckland New Zealand; 4 Tamaki Health Auckland New Zealand; 5 Auckland District Health Board Auckland New Zealand

**Keywords:** COVID-19, pandemic, mental health, mobile applications, apps, mHealth, coping skills, wellbeing, adolescent, young adult, coping

## Abstract

**Background:**

The COVID-19 pandemic is likely to increase anxiety and distress in young people worldwide. It is important to prioritize mental health during crisis events to mitigate the negative and often long-term effects of the crises on young people, families, and society. Mental health and well-being apps represent a scalable approach for improving psychological outcomes in young people and have potential to improve the equity of service access.

**Objective:**

The Whitu: 7 Ways in 7 Days well-being app was recently developed by our group to address the urgent need for innovative approaches to reach young New Zealanders who are struggling to cope with the COVID-19 pandemic. The aim of this study is twofold: to evaluate the acceptability of the prototype app and to examine the effectiveness of the refined app at improving mental and emotional well-being and reducing depression, anxiety, and stress in young people in New Zealand.

**Methods:**

A two-phase mixed methods study will be undertaken to achieve these aims. During the first phase, 20 young people aged 16-30 years (including those of Māori and Pacific ethnicity) will participate in a qualitative study to help refine the prototype app. During the second phase, 90 young people aged 16-30 years will participate in a randomized waitlist-controlled trial (RCT) to evaluate the efficacy of the refined Whitu app at 4 weeks and 3 months after baseline. Outcomes will be evaluated using validated web-based questionnaires at baseline, 4 weeks, and 3 months.

**Results:**

The study received ethics approval in May 2020, and recruitment for the focus groups commenced in June 2020. Recruitment for the RCT is expected to commence in October 2020. Participants for both study phases will be recruited via social media and web-based communities. Data collection for the RCT is expected to be completed by January 2021, and analyses are expected to be completed by March 2021. Linear mixed modelling will be used to determine between-group differences in psychological outcomes.

**Conclusions:**

There is an urgent need to develop culturally appropriate, scalable mental health interventions to address the psychological consequences of the COVID-19 pandemic. In this study, we will develop and test an evidence-based well-being app that, if effective, can be made available to all young people in New Zealand and internationally.

**Trial Registration:**

Australian New Zealand Clinical Trials Registry (ACTRN12620000516987); https://www.anzctr.org.au/Trial/Registration/TrialReview.aspx?id=379597.

**International Registered Report Identifier (IRRID):**

PRR1-10.2196/23716

## Introduction

### Background and Rationale

Prior to the COVID-19 pandemic, New Zealand young people, especially Māori (indigenous New Zealanders) and those of Pacific ethnicity, were already experiencing high rates of psychological distress and mental illness [1-4]. Crisis events, including pandemics, can result in significant mental health consequences both during and after the crisis, including increases in depression, anxiety, posttraumatic stress disorder, and suicide [5-8]. In the longer term, these problems can leave a damaging societal footprint, with markers such as increased exclusion or disengagement from education, academic underachievement, and unemployment [9]. It is likely that recent local stressors related to rapid lockdown, physical isolation, disrupted academic routines, and families’ financial insecurity will exacerbate pre-existing mental health issues and generate new ones, especially anxiety and depression in young people [10-12]. To minimize the immediate and future adverse psychological and social consequences of the pandemic, young people urgently need support to develop skills to maintain their well-being, address mental health issues early, and continue to build resilience during the coming months.

Over the past 20 years, rapidly evolving mobile technology has fostered the development of a range of eHealth interventions, including those designed to improve mental health, such as mental health apps [13]. Evidence has suggested that eHealth interventions for mental health conditions, such as depressive disorders, can be as effective as face-to-face therapies [14,15]. Acknowledging the effectiveness of eHealth interventions for people of all ages and the relatively good smartphone access in most developed countries, international organizations such as the Lancet Global Mental Health Group have highlighted the role of eHealth interventions in preventing and addressing common mental health issues in adults, young people, and children [16]. Key purported advantages of eHealth interventions include their flexibility of use, cost-effectiveness, and potential to increase equity of service access and reduce stigma [17]. These advantages are reflected by the willingness of people, especially “digital natives,” to use eHealth interventions [18].

Alongside these technological developments, evidence has been increasing that specific therapeutic modalities enhance mental health and well-being in children and young people and that these can effectively be delivered as eHealth interventions, such as cognitive behavioral therapy (CBT) [13,19] and psychoeducation [20]. Awareness is also growing that self-empowerment of well-being can improve the quality of care and outcomes for people experiencing mental health problems [21,22], and it has been suggested that interventions should equally aim to enhance well-being and attempt to reduce distress [23]. Digital interventions such as apps must be user-centered to produce the best outcomes for people [24,25]. Well-designed interventions can cost less to produce and lead to greater user satisfaction and content completion [25]. There are currently no evidence-based well-being apps specifically tailored for young people living in New Zealand to help them manage their psychological well-being during the COVID-19 pandemic.

This project has been designed to address the psychological needs of New Zealand young people aged 16-30 years, especially those of Māori and Pacific ethnicity, during the COVID-19 pandemic. At the start of the pandemic (in March 2020), a preliminary prototype app called Whitu: 7 Ways in 7 Days was rapidly developed by our group in response to our clinical concern for this cohort. *Whitu* is the Māori word for “seven,” and as its name suggests, the app includes seven modules that can be completed within a week to learn evidence-based coping skills based on our previous work using CBT, psychoeducation, and positive psychology techniques [26-28]. The seven modules and skills included in the app have all previously demonstrated efficacy for young people and as individual eHealth interventions: (1) identifying and rating emotions [20], (2) relaxation [19], (3) self-compassion [29,30], (4) gratitude [31,32], (5) staying connected [33,34], (6) physical care [35,36], and (7) goal setting [37,38].

The specific objectives of the current project are to refine the prototype evidence-based app for improving the well-being and mental health of New Zealand young people aged 16-30 years; ensure broad end-user and cultural acceptability of the app, particularly to Māori and Pacific young people; and demonstrate the preliminary clinical effectiveness of the app via objective outcome measurement during a randomized waitlist-controlled trial (RCT).

## Methods

### Study Design

A two-phase, mixed-methods design will be employed. During the first phase, a qualitative study will be conducted with users of the prototype app, and the app will be refined on the basis of their feedback. During the second phase, an RCT will be conducted according to CONSORT (Consolidated Standards of Reporting Trials) guidelines [39].

### Phase 1: Qualitative Study

#### Participants

Approximately 20 young people from New Zealand aged between 16 and 30 years will be recruited during June to August 2020 from web-based communities and groups (eg, Facebook, Instagram, Tuakana-teina/Māori student mentorship programs) to help refine the prototype app design and content. A series of five focus groups will be conducted to collect feedback, with three to six people per group. We will recruit at least ten Māori and Pacific young people to ensure the app appeals to these audiences and is culturally appropriate.

#### Procedures

Recruitment will be conducted and informed consent will be obtained on the internet over a secure website, Research Electronic Data Capture (REDCap) [40,41]. Prior to attending the focus groups, participants will be asked to provide demographic variables, including age, sex, ethnicity, pre-existing mental health conditions, and experience of using well-being and mental health apps, via self-report questionnaires on REDCap. Participants will also be asked to download and use the prototype app for approximately one week prior to attending the focus groups.

The focus groups will last between 1.5 and 2 hours and will be conducted via Zoom, with two focus groups including only Māori and Pacific young people. To ensure culturally appropriate processes and participant comfort, these focus groups will be facilitated by our Māori and Pacific researchers (EM and NC). Ideas for integrating wider Māori and Pacific views of mental well-being will be explored, and the cultural appropriateness of Māori and Pacific design motifs will be assessed.

During the focus groups, participants will provide audio-recorded feedback on their experiences of using all seven modules. All participants will receive a voucher for NZ$40 (US $26.36) for attending the focus groups.

#### Data Analysis

Participants’ qualitative data will be audio-recorded and transcribed. The transcribed data will be analyzed using directed content analysis [42], a qualitative approach that is well suited for focus groups or interviews where predetermined concepts or categories are examined (eg, usability and acceptability of the different functions and content of the app). The coding scheme will be partly based on the user version of the Mobile Application Rating Scale (uMARS) [43] domains (Engagement, Functionality, Aesthetics, and Information), and the cultural acceptability of the scheme will also be explored by EM and NC. The data will be coded and analyzed independently by at least two members of the research team. Any discrepancies in coding will be resolved by consulting the wider research team.

### Phase 2: Randomized Waitlist-Controlled Trial

#### Participants

Participants will be New Zealand residents between the ages of 16 and 30 years. Recruitment is expected to commence in October 2020 via social media (Facebook, Instagram) as well as on other New Zealand–based web-based communities. Young people who participated in phase 1 (qualitative study) will not be eligible to participate in the RCT. Participants will receive a NZ$40 voucher (US $26.36) for taking part in the RCT. Participants who are currently receiving mental health treatment (including using a mental health app) are not eligible to participate.

#### Procedures

**Figure 1:**
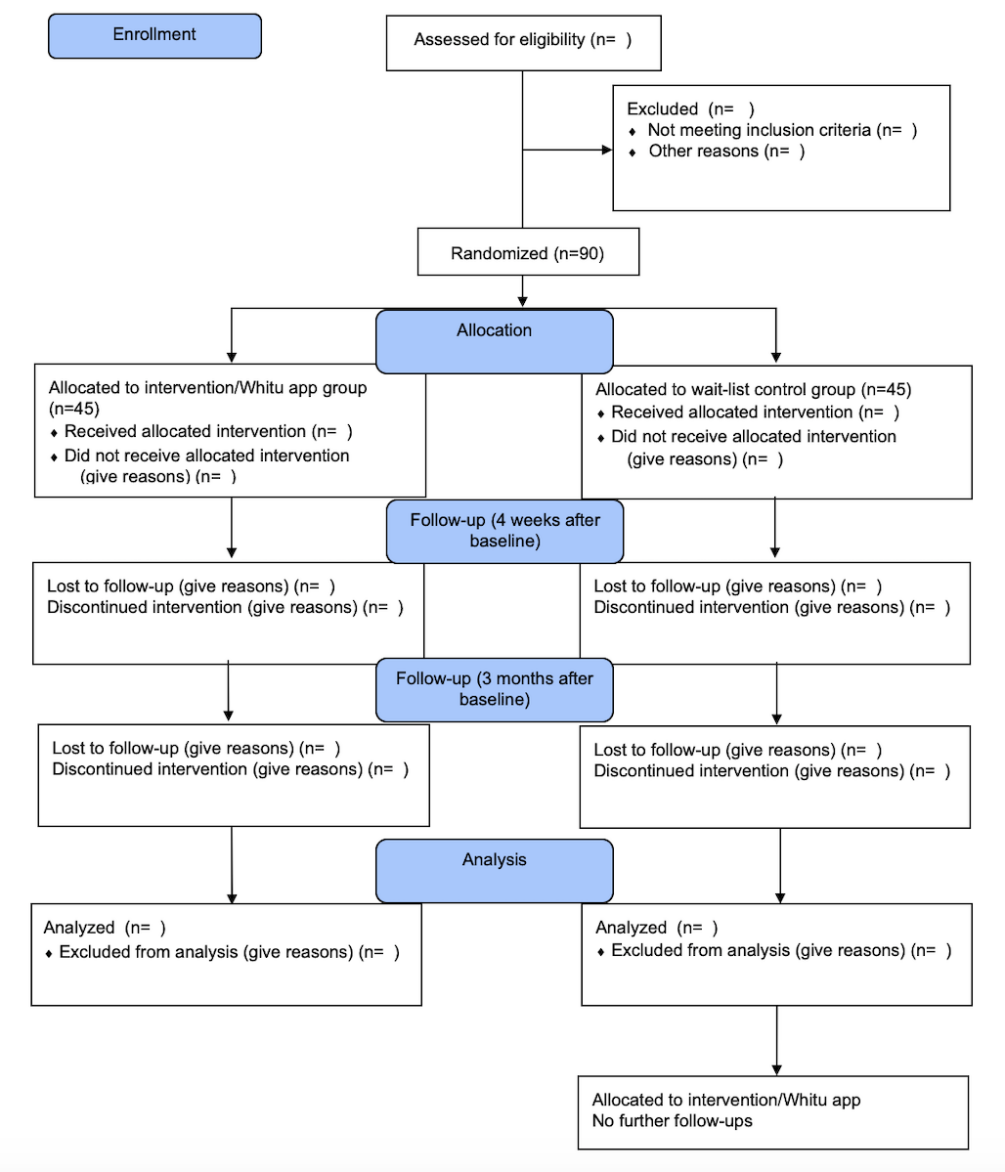
CONSORT (Consolidated Standards of Reporting Trials) flow diagram of participant recruitment, randomization, and attrition.

Participants will be recruited from web-based communities (eg, social media) by an advertisement or flyer with a link to the REDCap site. On REDCap, interested participants will be screened for eligibility. If eligible, they can read and download the Participant Information Sheet, provide web-based consent, and complete the baseline questionnaires once they have consented. Once participants have consented and completed the baseline questionnaires, they will be randomized into either the intervention group (Whitu app) or wait-list control group. Randomization will occur using REDCap’s randomization module. Participants and research staff will not be blinded to treatment allocation. The wait-list control group will receive the app 3 months after the initial app group. Figure 1 shows the CONSORT flow diagram.

After randomization, the wait-list group will be informed that they are in the wait-list control group on REDCap. The intervention group will be shown a link and instructions on how to download the Whitu app on REDCap. During the first four weeks, participants in the intervention group will receive 2 emails reminding them to complete the app modules and an SMS text message asking them to confirm whether they have been able to download the app. Four weeks after completing the baseline questionnaires, participants in both groups will receive an email with a link to the first follow-up questionnaire on REDCap. Three months after baseline, both groups will receive an email with the link to the final follow-up assessment. After the wait-list control group completes the 3-month assessment, REDCap will send each group member an automated email with instructions on how to download the Whitu app. We will not follow up with the wait-list control group after they receive the app at the 3-month time-point.

All participant outcome data (eg, demographic data and psychological outcomes) will be collected via REDCap. We will not collect any information via the app, and any user input (eg, first name, responses to exercises) will only be stored locally on the user’s device. Only the Principal Investigators and research assistants (AS, HT, AB, and DL) will have access to the participant data collected over REDCap. When the data are exported from REDCap, all data will be deidentified and stored separately from the participants’ information.

### Power Calculation

A previous study of a web-based positive psychology intervention for mildly depressed adults [44] found a between-group improvement in well-being (effect size of *f*=0.155) using the World Health Organization-Five Well-Being Index (WHO-5) [45]. Using G*Power [46], we calculated that the minimum required sample size to detect an effect size of *f*=0.155 using a mixed analysis of variance (ANOVA) including within-subject (3 time points) and between-subject (2 groups) effects with 90% power and at a 2-sided 5% significance level was 90 participants (45 per treatment arm). To ensure cultural acceptability, we will aim to recruit at least 40% Māori and Pacific young people (n=36).

### Ethical Approval and Trial Registration

The study received ethics approval from the University of Auckland Human Participant Ethics Committee on June 18, 2020 (Ethics Committee reference: 024542). The protocol for the RCT was prospectively registered with the Australian New Zealand Clinical Trials Registry on April 17, 2020 (ACTRN12620000516987).

### Intervention: Whitu: 7 Ways in 7 Days

The intervention has been developed as a cross-platform app; therefore, it will function on both Android and iOS operating systems. Data or internet connectivity is required to stream the informational videos contained in each module. The app is free to download. No user information or app analytics data are collected or stored over the internet. Any data input by the user are stored locally on the user’s device in an unencrypted SQLite database. These data can be safely deleted at any time by the user via the system settings on the device or by deleting the app.

Evidence shows that young people often do not use health apps for long periods; for example, one review of 93 health apps found that only 3.9% of people who downloaded these apps used them for a median of 15 days [47], and another review of 10 self-help interventions for anxiety and depression found that only 0.5% to 28.7% of users completed the interventions [48]. Therefore, the Whitu app has been purposely designed to be completed over the course of one week. The user receives daily push notifications reminding them to complete at least one module per day and to practice preferred exercises from previous modules. Many CBT-based, psychoeducational, and positive psychology interventions focus on teaching a repertoire of coping strategies and techniques to allow users to choose from a broad range of strategies and discover which ones work best for them individually. In a similar fashion, our aim in offering seven modules is to ask participants to try all the modules over the course of one week and keep practicing the strategies that work best for them. The seven modules are described in detail in Table 1.

**Table 1:** The seven modules included in the Whitu: 7 Ways in 7 Days app.

Module	Description
Module 1: Feel	The first module acknowledges that young people may be feeling low and struggling with negative emotions due to COVID-19. The module introduces the concept of identifying and monitoring emotions and recognizing adaptive and maladaptive coping skills. Exercises include recognizing and rating recent emotions as well as grouping coping skills into adaptive and maladaptive categories.
Module 2: Relax	The second module recognizes the uncertainty and stress that young people may be feeling due to the COVID-19 pandemic. The module introduces common relaxation techniques to manage stressful situations. Exercises including deep breathing, progressive muscle relaxation, and visualization.
Module 3: Be kind to yourself	The third module introduces the concept self-compassion and includes a short check-in meditation. The module then asks participants to be mindful of a hard feeling they have had in the past week, reflect on whether friends and family have felt the same, and write self-kindness statements.
Module 4: Be thankful	The fourth module introduces the concept of gratitude and how it is linked to positive well-being. This module encourages grateful contemplation and action by asking users to list things they are thankful for daily and in different situations and to keep a written or photographic record of things they are thankful for.
Module 5: Connect	The fifth module recognizes the negative impact that lockdowns as well as physical distancing can have on relationships. The module introduces the importance of social connection for well-being. It encourages participants to identify important people in their lives and to practice ways of staying connected with them.
Module 6: Look after your body	The sixth module discusses how the COVID-19 situation makes it more difficult to stay active and look after our bodies. This module introduces how eating well, moving regularly, and getting rest support psychological well-being. Information is provided about the key food groups, benefits of physical activity, and sleep. Participants are encouraged to plan how to improve their diet, increase their physical activity, and get more sleep.
Module 7: Set goals	The seventh module acknowledges that the COVID-19 pandemic has likely interrupted users’ routines and made it harder to set healthy goals. This module introduces goal setting and guides the user to set their own specific, measurable, achievable, realistic, and time-based (SMART) goals. Explores benefits and barriers to setting different goals and helps the user build confidence by identifying various factors that impact the process of achieving their particular goal.

#### Onboarding

**Figure 2:**
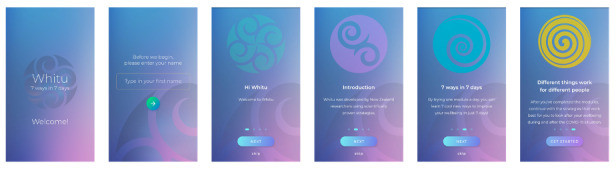
Screenshots of the onboarding process of the Whitu: 7 Ways in 7 Days app.

When the user first opens the app, they are presented with a splash screen containing the app logo, the app title, and a welcome message. This is followed by a screen where the user can input their name. This name is used to personalize messages and exercises throughout the app. The user is then presented with four Onboarding screens, including a welcome message and a brief explanation of the features of the app (see Figure 2).

#### Badges

Users earn badges as they complete modules and revision exercises. The aesthetics of the badges are inspired by Māori and Pacific design motifs. Earned badges will appear in color on the Badges screen, while unearned badges appear greyed out. Users can collect a total of 20 badges.

#### Videos

Each module contains at least one informative video that provides a graphical and audio description of its content. The videos are narrated by two characters, Ana and Ben.

#### Exercises

**Figure 3:**
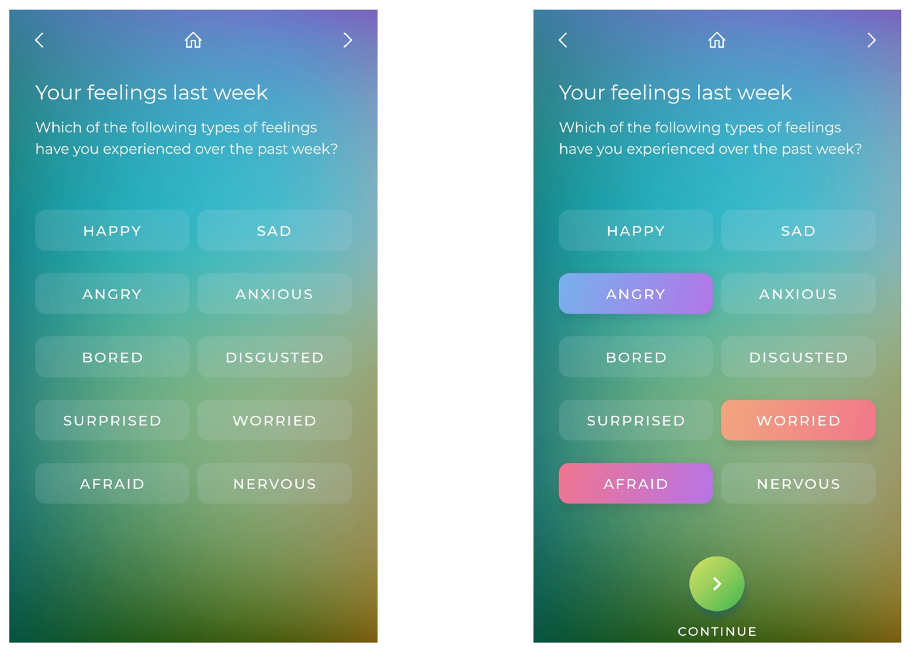
Screenshots of the Identifying Emotions exercise from Module 1 of the Whitu: 7 Ways in 7 Days app.

Each module contains one or more exercises that demonstrate the well-being concepts and techniques described in the videos. For example, in Exercise 1 of Module 1, users are asked to identify and select various emotions they may have experienced over the past week (Figure 3).

#### Completed Modules

**Figure 4:**
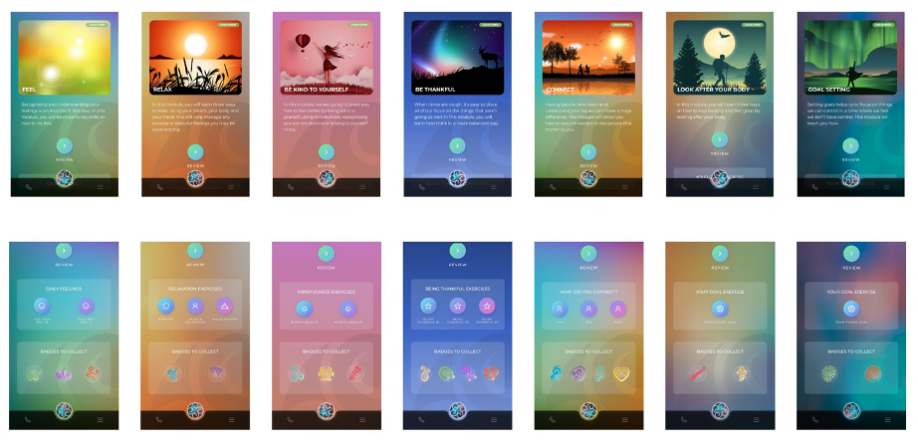
Screenshots of the seven modules and associated badges of the Whitu: 7 Ways in 7 Days app.

When users have viewed all videos and completed all exercises in a module, they are able to review the content of the module or carry out revision exercises intended to refresh and reinforce the message of each module. Users earn badges for each revision exercise they complete (see Figure 4).

#### Revision Exercises

Each module has one or more associated revision exercises that only become available after the module is completed. For some modules, the same revision exercise can be repeated across multiple days. For example, the revision exercise for Module 1 is a condensed version of the main module content that users can use to track their feelings over two days.

### Outcome Measures

Outcome measures will be assessed at baseline, 4 weeks after baseline, and 3 months after baseline. Demographic data, including sex, age, ethnicity, and pre-existing mental health conditions, will be collected at baseline via self-report on REDCap. All the outcome measures listed below are brief and have acceptable reliability and validity. Completing the questionnaires at each time point will take approximately 20-30 minutes.

#### Primary Outcomes

##### 1. Emotional Well-Being

This outcome will be assessed via the WHO-5 [45]. The WHO-5 is a 5-item scale that assesses positive emotional well-being (eg, “I have felt cheerful and in good spirits”). Participants are asked to indicate the extent to which each item matches how they have been feeling over the past two weeks on a 6-point Likert scale ranging from 0 (“at no time”) to 5 (“all of the time”). The final score is calculated by summing the five responses and multiplying the total by four. This gives a percentage score ranging from 0-100, with 0 representing the worst possible well-being and 100 representing the best possible well-being. The internal consistency of the WHO-5 demonstrates good reliability (α=.84) in children and adolescents [49].

##### 2. Mental Well-Being

This outcome will be assessed by the Short Warwick-Edinburgh Mental Well-being Scale (SWEMWBS) [50,51]. The SWEMWBS is a short version of the 14-item Warwick-Edinburgh Mental Well-Being Scale (WEMWBS), which is comprised of positively worded items measuring different aspects of positive mental health [41]. The SWEMWBS is a 7-item scale that asks participants to rate their experience of a range of thoughts and feelings (eg, “I’ve been dealing with problems well”) over the last two weeks on a 5-point Likert scale ranging from 1 (“none of the time”) to 5 (“all of the time”). The score is calculated by summing individual ratings and transforming the total into a metric score using a transformation provided by the scale authors. Scores range from 7 to 35, with higher scores indicating greater positive mental well-being. The original scale has demonstrated good content validity and is correlated with other mental health and well-being measures [50]. The short version has demonstrated similar reliability and validity to the full version (α=.84) and is suitable to be used by adolescents [52,53].

#### Secondary Outcomes

##### 1. Depression

This outcome will be assessed by the short version of the Centre for Epidemiological Studies Depression Scale (CES-D) [54]. The CES-D is a 20-item scale designed to measure depressive symptoms in the general population. Participants are asked to rate on a 4-point Likert scale how often they have experienced a variety of different feelings or behaviors (eg, “My sleep was restless”), ranging from 0, rarely or none of the time (less than 1 day) to 3, most or all of the time (5-7 days). Scores are calculated by reverse scoring positive items and summing item totals, yielding a total score between 0 and 60. Higher scores indicate more depressive symptoms. The scale has demonstrated excellent internal consistency (α=.85) and demonstrates high correlations with other depression measures [54].

##### 2. Anxiety

This outcome will be assessed by the Generalized Anxiety Disorder seven item scale (GAD-7) [55]. The GAD-7 is a 7-item self-report scale assessing how often participants experience different symptoms of anxiety (eg, “feeling nervous, anxious, or on edge”). Items are rated on a 4-point Likert scale including 0, not at all; 1, several days; 2, more than half the days; and 3, nearly every day, yielding one total score. Scores of 5, 10, and 15 represent cutoffs for mild, moderate, and severe anxiety, respectively. This scale has been demonstrated to be capable of identifying probable cases of generalized anxiety disorder. It demonstrates excellent reliability (α=.92) and validity [55] in a general population [56] and in adolescents [57].

##### 3. Stress

This outcome will be assessed by the 10-item Perceived Stress Scale (PSS-10) [58,59]. The PSS-10 is a 10-item scale that assesses the extent to which individuals have felt a range of stressors over the last month. Participants are asked to rate how often they experienced different stressful thoughts and feelings (eg, “In the last month, how often have you felt that you were unable to control the important things in your life?”) on a 5-point Likert scale ranging from 0, never, to 4, very often. A total score is calculated by reverse scoring items 4, 5, 7, and 8 and summing all ten of the responses. Scores below 13 are considered to indicate low stress, scores between 14 and 26 are considered to indicate moderate stress, and scores between 27 and 40 are considered to indicate high stress. The PSS-10 scale has superior psychometric properties relative to other existing perceived stress scales and has demonstrated good reliability and validity [60].

##### 4. Self-Compassion

This outcome will be assessed using the Self-Compassion Scale–Short Form (SCS-SF) [61].The SCS-SF is an alternative to the long form Self-Compassion Scale [62]. The SCS-SF comprises 12 items that ask participants to rate how they typically act toward themselves in different situations (eg, “When I’m going through a very hard time, I give myself the caring and tenderness I need”) on a 5-point Likert scale ranging from 1, almost never, to 5, almost always. The SCS-SF has six subscales (overidentification, self-kindness, mindfulness, isolation, common humanity, and self-judgement) comprising two questions each. Scores are calculated by reverse scoring the negative subscale items, calculating the mean for each subscale, and computing a total mean. Higher scores indicate greater levels of self-compassion. The SCS-SF demonstrates good reliability (α>.86), is highly correlated with the long form scale [49], and has shown adequate reliability in an adolescent sample [63].

##### 5. Sleep

This outcome will be assessed using a single-item Sleep Quality Scale (SQS) [64]. This single-item questionnaire (“During the past 7 days, how would you rate your sleep quality overall?”) is assessed on an 11-point visual analog scale (VAS) from 0-10. The VAS scores are anchored with 0, terrible; 1-3, poor; 4-6, fair; 7-9, good; and 10, excellent. The SQS has demonstrated excellent concurrent and convergent validity with other lengthier sleep scales and has been deemed to be effective in determining clinically meaningful changes in sleep quality [64].

##### 6. User Engagement

This outcome will be assessed via two subscales (App Subjective Quality, Perceived Impact) of the end-user version of the uMARS [65]. The uMARS is a self-report scale designed to assess the quality of health apps. The Subjective Quality score for the app is derived from four items that determine user experience (eg, “Would you recommend this app to people who might benefit from it?”). These items are scored on a 5-point Likert scale ranging from 1 to 5; however, each has different anchors. Scores from this subscale can be reported individually or as a mean total. The Perceived Impact score consists of six items that measure the impact of using the app on knowledge, attitudes, and intentions. The six items are reported as individual items and measured on a Likert scale ranging from 1 to 5 (1, strongly disagree; 5, strongly agree). The uMARS demonstrates good internal reliability overall (α=.90), and the two subscales demonstrate good to moderate reliability (α=.71 and .80) [43]. The scale also contains an open-ended question that asks: “Do you have any further comments about the app?”

In addition to the uMARS, participants will also be asked the following questions about their use of the Whitu app: (1) How many modules did you complete? (2) On how many days did you use the app? (3) What module was the most useful? Why? (4) What did you like about the app? (5) How can we make the app better for young people in the future? and (7) Did you experience any technical difficulties with the app? If yes, what happened?

#### Hypotheses and Statistical Analyses

It is predicted that the Whitu app group will demonstrate improved well-being (increased emotional and mental well-being), increased self-compassion, and improved sleep at 4-week and 3-month follow-up compared to the wait-list control group. We also hypothesize that the Whitu app group will demonstrate decreased stress, depression, and anxiety at 4-week and 3-month follow-up compared to the wait-list control group.

Data will be analyzed in SPSS (IBM Corporation) or SAS (SAS Institute). Prior to any data analysis, the data will be tested for violations of statistical assumptions and screened for errors and outliers. If parametric assumptions are not met due to nonnormality, transformations or nonparametric tests will be used. Pearson correlations (or Spearman ρ if nonnormal) will be used to explore the relationships between the outcome measures and demographic characteristics and other psychosocial factors. ANOVAs or Kruskal-Wallis tests (if nonnormal) will be used to explore associations between categorical variables and outcome measures. Linear mixed models will be used to determine whether changes in psychological outcomes are the result of the interaction between the intervention group and time, with post-hoc tests to assess pairwise comparisons of groups at each time point and within-group changes over time. Linear mixed models also allow for missing data, as they enable all participants with baseline data to be included in the analysis. We will also examine possible 3-way interactions with age, sex, or ethnicity (eg, age*group*time). Means, SDs, and CIs will be presented with the analysis. Data will be subjected to both intention-to-treat and per-protocol analyses. Per-protocol analyses will include participants who report completing all seven modules. Study results will be disseminated through peer-reviewed journals and conferences.

## Discussion

### General Considerations

Through this mixed-methods study, we hope to develop a coping skills app for young people in New Zealand aged 16-30 years to use during and immediately following the COVID-19 pandemic and to provide preliminary evidence of its acceptability and effectiveness at improving well-being and other mental health outcomes. As young people of Māori and Pacific ethnicity are already at greater risk of developing psychological problems such as anxiety and depression, we hope the app is particularly appealing and useful for young people in these groups. As the app is based on proven strategies and techniques for improving psychological well-being, we hope it will also be of longer-term value to its users.

Limitations of this study may include low adherence and attrition, which we have attempted to minimize by framing the intervention as a resource that can be completed within seven days. Another possible limitation is that we are only assessing self-reported user engagement. In future prototypes, we hope to also include app analytics to explore more objective measures of user engagement. Lastly, as the app is tailored to New Zealand youth aged 16-30 years, we will not be able to generalize our findings to people younger than 16 years or those outside New Zealand. However, if the app is effective in improving well-being, we hope to conduct a larger and more definitive study in the future that includes younger participants and those outside New Zealand.

### Conclusions

There is a pressing need for mental health professionals to create appropriate resources and interventions to improve well-being and help prevent mental health deterioration due to the COVID-19 pandemic. Our study builds on our previous research and addresses the clear needs to develop a mental health toolkit to help New Zealanders cope with the COVID-19 pandemic [66] and to develop a resource that is cost-effective and scalable and that reduces mental health inequities.

We predict that use of the app will lead to maintenance of well-being and reduced rates of anxiety and depression throughout and immediately following the COVID-19 pandemic. In the longer-term, we hope that the skills learned via the app will also result in reduced need to access mental health services for COVID-19–related issues. Economically, improved well-being, reduced mental health problems, and maintenance of usual activities are likely to result in less societal workforce disruption and loss of productivity. This project also provides opportunities for future research collaboration to evaluate this scalable and evidence-based app with an international audience.

## Appendix

Multimedia Appendix 1 Peer review report.

## Abbreviations

ANOVA: analysis of variance
CBT: cognitive behavior therapy
CES-D: Centre for Epidemiological Studies Depression Scale
CONSORT: Consolidated Standards of Reporting Trials
PSS-10: 10-item Perceived Stress Scale
RCT: randomized wait-list controlled trial
REDCap: Research Electronic Data Capture
SCS-SF: Self-Compassion Scale–Short Form
SMART: specific, measurable, achievable, realistic, time-based
SQS: Sleep Quality Scale
SWEMWBS: Short Warwick-Edinburgh Mental Well-being Scale
uMARS: user version of the Mobile Application Rating Scale
VAS: visual analog scale
WHO-5: World Health Organization-Five Well-Being Index

## References

[1] Crengle S, Clark T, Robinson E, Bullen P, Dyson B, Denny S, Fleming T, Fortune S, Peiris-John R, Utter J, Rossen F, Sheridan J, Teevale T, The Adolescent Health Research Group (2013). The health and wellbeing of Māori New Zealand secondary school students in 2012. Te Ara Whakapiki Taitamariki: Youth’12.

[2] Clark T, Robinson E, Crengle S, Herd R, Grant S, Denny S (2008). Te Ara Whakapiki Taitamariki. Youth’07: The Health and Wellbeing Survey of Secondary School Students in New Zealand. Results for Māori Young People.

[3] Rasanathan K, Ameratunga S, Chen J, Robinson E, Young W, Wong G, Garrett N, Watson PD (2006). A health profile of young Asian New Zealanders who attend secondary school: findings from Youth2000.

[4] Ministry OH (2016). Understanding suicide in New Zealand. New Zealand Ministry of Health.

[5] Norris FH (2005). Range, Magnitude, and Duration of the Effects of Disasters on Mental Health: Review Update 2005. ResearchGate.

[6] Douglas PK, Douglas DB, Harrigan DC, Douglas KM (2009). Preparing for pandemic influenza and its aftermath: mental health issues considered. Int J Emerg Ment Health.

[7] North CS, Pfefferbaum B (2013). Mental health response to community disasters: a systematic review. JAMA.

[8] Wheaton MG, Abramowitz JS, Berman NC, Fabricant LE, Olatunji BO (2011). Psychological Predictors of Anxiety in Response to the H1N1 (Swine Flu) Pandemic. Cogn Ther Res.

[9] Khan L (2016). Missed opportunities: A review of recent evidence into children and young people's mental health. Centre for Mental Health.

[10] (2020). Coronavirus: Impact on Young People with Mental Health Needs. YoungMinds.

[11] Crawley E, Loades M, Feder G, Logan S, Redwood S, Macleod J (2020). Wider collateral damage to children in the UK because of the social distancing measures designed to reduce the impact of COVID-19 in adults. BMJ Paediatr Open.

[12] Lee J (2020). Mental health effects of school closures during COVID-19. Lancet Child Adolesc Health.

[13] Hollis C, Falconer CJ, Martin JL, Whittington C, Stockton S, Glazebrook C, Davies EB (2017). Annual Research Review: Digital health interventions for children and young people with mental health problems - a systematic and meta-review. J Child Psychol Psychiatry.

[14] Merry SN, Stasiak K, Shepherd M, Frampton C, Fleming T, Lucassen MFG (2012). The effectiveness of SPARX, a computerised self help intervention for adolescents seeking help for depression: randomised controlled non-inferiority trial. BMJ.

[15] Luo C, Sanger N, Singhal N, Pattrick K, Shams I, Shahid H, Hoang P, Schmidt J, Lee J, Haber S, Puckering M, Buchanan N, Lee P, Ng K, Sun S, Kheyson S, Chung DCY, Sanger S, Thabane L, Samaan Z (2020). A comparison of electronically-delivered and face to face cognitive behavioural therapies in depressive disorders: A systematic review and meta-analysis. EClinicalMedicine.

[16] Lancet Global Mental Health Group (2007). Scale up services for mental disorders: a call for action. Lancet.

[17] Schröder J, Berger T, Westermann S, Klein JP, Moritz S (2016). Internet interventions for depression: new developments. Dialogues Clin Neurosci.

[18] Torous J, Chan SR, Yee-Marie Tan S, Behrens J, Mathew I, Conrad EJ, Hinton L, Yellowlees P, Keshavan M (2014). Patient Smartphone Ownership and Interest in Mobile Apps to Monitor Symptoms of Mental Health Conditions: A Survey in Four Geographically Distinct Psychiatric Clinics. JMIR Ment Health.

[19] Spence SH, Donovan CL, March S, Gamble A, Anderson RE, Prosser S, Kenardy J (2011). A randomized controlled trial of online versus clinic-based CBT for adolescent anxiety. J Consult Clin Psychol.

[20] Taylor-Rodgers E, Batterham PJ (2014). Evaluation of an online psychoeducation intervention to promote mental health help seeking attitudes and intentions among young adults: randomised controlled trial. J Affect Disord.

[21] (2016). Going digital to deliver wellbeing services to young people? Insights from e-tools supporting youth mental health and parenting. Superu.

[22] Sin NL, Lyubomirsky S (2009). Enhancing well-being and alleviating depressive symptoms with positive psychology interventions: a practice-friendly meta-analysis. J Clin Psychol.

[23] Keyes CLM (2007). Promoting and protecting mental health as flourishing: A complementary strategy for improving national mental health. Am Psychol.

[24] Yardley L, Morrison L, Bradbury K, Muller I (2015). The person-based approach to intervention development: application to digital health-related behavior change interventions. J Med Internet Res.

[25] Thabrew H, Fleming T, Hetrick S, Merry S (2018). Co-design of eHealth Interventions With Children and Young People. Front Psychiatry.

[26] Serlachius AS, Scratch SE, Northam EA, Frydenberg E, Lee KJ, Cameron FJ (2016). A randomized controlled trial of cognitive behaviour therapy to improve glycaemic control and psychosocial wellbeing in adolescents with type 1 diabetes. J Health Psychol.

[27] Schache KR, Hofman PL, Serlachius AS (2020). A pilot randomized controlled trial of a gratitude intervention for adolescents with Type 1 diabetes. Diabet Med.

[28] Boggiss AL, Consedine NS, Jefferies C, Bluth K, Hofman PL, Serlachius AS (2020). Protocol for a feasibility study: a brief self-compassion intervention for adolescents with type 1 diabetes and disordered eating. BMJ Open.

[29] Neff KD, Germer CK (2013). A pilot study and randomized controlled trial of the mindful self-compassion program. J Clin Psychol.

[30] Finlay-Jones A, Kane R, Rees C (2017). Self-Compassion Online: A Pilot Study of an Internet-Based Self-Compassion Cultivation Program for Psychology Trainees. J Clin Psychol.

[31] Froh JJ, Yurkewicz C, Kashdan TB (2009). Gratitude and subjective well-being in early adolescence: examining gender differences. J Adolesc.

[32] Baños RM, Etchemendy E, Mira A, Riva G, Gaggioli A, Botella C (2017). Online Positive Interventions to Promote Well-being and Resilience in the Adolescent Population: A Narrative Review. Front Psychiatry.

[33] Barrera JM, Prelow H, Cicchetti D, Rappaport J, Sandler I, Weissberg RP (2000). Interventions to promote social support in children and adolescents. The promotion of wellness in children and adolescents.

[34] McLaughlin M, Nam Y, Gould J, Pade C, Meeske KA, Ruccione KS, Fulk J (2012). A videosharing social networking intervention for young adult cancer survivors. Comput Hum Behav.

[35] Oosterveen E, Tzelepis F, Ashton L, Hutchesson MJ (2017). A systematic review of eHealth behavioral interventions targeting smoking, nutrition, alcohol, physical activity and/or obesity for young adults. Prev Med.

[36] Blake MJ, Blake LM, Schwartz O, Raniti M, Waloszek JM, Murray G, Simmons JG, Landau E, Dahl RE, McMakin DL, Dudgeon P, Trinder J, Allen NB (2018). Who benefits from adolescent sleep interventions? Moderators of treatment efficacy in a randomized controlled trial of a cognitive-behavioral and mindfulness-based group sleep intervention for at-risk adolescents. J Child Psychol Psychiatry.

[37] Shilts MK, Horowitz M, Townsend MS (2004). An Innovative Approach to Goal Setting for Adolescents: Guided Goal Setting. Journal of Nutrition Education and Behavior.

[38] Cushing CC, Steele RG (2010). A meta-analytic review of eHealth interventions for pediatric health promoting and maintaining behaviors. J Pediatr Psychol.

[39] Schulz K, Altman D, Moher D, CONSORT Group (2010). CONSORT 2010 Statement: updated guidelines for reporting parallel group randomised trials. Trials.

[40] Harris PA, Taylor R, Thielke R, Payne J, Gonzalez N, Conde JG (2009). Research electronic data capture (REDCap)--a metadata-driven methodology and workflow process for providing translational research informatics support. J Biomed Inform.

[41] Harris P, Taylor R, Minor B, Elliott V, Fernandez M, O'Neal L, McLeod L, Delacqua G, Delacqua F, Kirby J, Duda SN, REDCap Consortium (2019). The REDCap consortium: Building an international community of software platform partners. J Biomed Inform.

[42] Hsieh H, Shannon SE (2005). Three approaches to qualitative content analysis. Qual Health Res.

[43] Stoyanov SR, Hides L, Kavanagh DJ, Wilson H (2016). Development and Validation of the User Version of the Mobile Application Rating Scale (uMARS). JMIR mHealth uHealth.

[44] Bolier L, Haverman M, Kramer J, Westerhof GJ, Riper H, Walburg JA, Boon B, Bohlmeijer E (2013). An Internet-based intervention to promote mental fitness for mildly depressed adults: randomized controlled trial. J Med Internet Res.

[45] Bech P, Olsen LR, Kjoller M, Rasmussen NK (2003). Measuring well-being rather than the absence of distress symptoms: a comparison of the SF-36 Mental Health subscale and the WHO-Five Well-Being Scale. Int J Methods Psychiatr Res.

[46] Faul F, Erdfelder E, Lang A, Buchner A (2007). G*Power 3: a flexible statistical power analysis program for the social, behavioral, and biomedical sciences. Behav Res Methods.

[47] Baumel A, Muench F, Edan S, Kane JM (2019). Objective User Engagement With Mental Health Apps: Systematic Search and Panel-Based Usage Analysis. J Med Internet Res.

[48] Fleming T, Bavin L, Lucassen M, Stasiak K, Hopkins S, Merry S (2018). Beyond the Trial: Systematic Review of Real-World Uptake and Engagement With Digital Self-Help Interventions for Depression, Low Mood, or Anxiety. J Med Internet Res.

[49] Allgaier A, Pietsch K, Frühe B, Prast E, Sigl-Glöckner J, Schulte-Körne G (2012). Depression in pediatric care: is the WHO-Five Well-Being Index a valid screening instrument for children and adolescents?. Gen Hosp Psychiatry.

[50] Tennant R, Hiller L, Fishwick R, Platt S, Joseph S, Weich S, Parkinson J, Secker J, Stewart-Brown S (2007). The Warwick-Edinburgh Mental Well-being Scale (WEMWBS): development and UK validation. Health Qual Life Outcomes.

[51] Ng Fat L, Scholes S, Boniface S, Mindell J, Stewart-Brown S (2017). Evaluating and establishing national norms for mental wellbeing using the short Warwick-Edinburgh Mental Well-being Scale (SWEMWBS): findings from the Health Survey for England. Qual Life Res.

[52] McKay MT, Andretta JR (2017). Evidence for the Psychometric Validity, Internal Consistency and Measurement Invariance of Warwick Edinburgh Mental Well-being Scale Scores in Scottish and Irish Adolescents. Psychiatry Res.

[53] Ringdal R, Bradley Eilertsen M, Bjørnsen HN, Espnes GA, Moksnes UK (2018). Validation of two versions of the Warwick-Edinburgh Mental Well-Being Scale among Norwegian adolescents. Scand J Public Health.

[54] Radloff LS (2016). The CES-D Scale: A Self-Report Depression Scale for Research in the General Population. Appl Psychol Meas.

[55] Spitzer RL, Kroenke K, Williams JBW, Löwe B (2006). A brief measure for assessing generalized anxiety disorder: the GAD-7. Arch Intern Med.

[56] Löwe B, Decker O, Müller S, Brähler E, Schellberg D, Herzog W, Herzberg PY (2008). Validation and Standardization of the Generalized Anxiety Disorder Screener (GAD-7) in the General Population. Med Care.

[57] Mossman S, Luft M, Schroeder H, Varney S, Fleck D, Barzman D (2017). The Generalized Anxiety Disorder 7-item (GAD-7) scale in adolescents with generalized anxiety disorderignal detection and validation. Ann Clin Psychiatry.

[58] Cohen S, Kamarck T, Mermelstein R (1983). A Global Measure of Perceived Stress. Journal of Health and Social Behavior.

[59] Cohen S, Kamarck T, Mermelstein R (1994). Perceived Stress Scale. APA PsycTests.

[60] Lee E (2012). Review of the psychometric evidence of the perceived stress scale. Asian Nurs Res (Korean Soc Nurs Sci).

[61] Raes F, Pommier E, Neff KD, Van Gucht D (2011). Construction and factorial validation of a short form of the Self-Compassion Scale. Clin Psychol Psychother.

[62] NEFF KD (2003). The Development and Validation of a Scale to Measure Self-Compassion. Self Identity.

[63] Bluth K, Gaylord SA, Campo RA, Mullarkey MC, Hobbs L (2016). Making Friends With Yourself: A Mixed Methods Pilot Study of a Mindful Self-Compassion Program for Adolescents. Mindfulness.

[64] Snyder E, Cai B, DeMuro C, Morrison MF, Ball W (2018). A New Single-Item Sleep Quality Scale: Results of Psychometric Evaluation in Patients With Chronic Primary Insomnia and Depression. J Clin Sleep Med.

[65] Stoyanov SR, Hides L, Kavanagh DJ, Wilson H (2016). Development and Validation of the User Version of the Mobile Application Rating Scale (uMARS). JMIR mHealth uHealth.

[66] The S (2020). Covid-19 live updates, April 7: Mental health resource Getting Through Together now available. The Spinoff.

