# Supplementary material for: Coping Skills Mobile App to Support the Emotional Well-Being of Young People During the COVID-19 Pandemic: Protocol for a Mixed Methods Study
Source: JMIR Res Protoc. 2020 Oct 8;9(10):e23716. doi: 10.2196/23716 (PMC7546866; doi:10.2196/23716)
Supplement: Multimedia Appendix 1 [file resprot_v9i10e23716_app1.pdf]

Auckland Medical
Research Foundation
est. 1955

24 August 2020

PO Box 110139, Auckland Hospital,
Auckland 1148

Tel: +64 9 923 1701
Fax: +64 9 362 0458
Email: amrf@medicalresearch.org.nz

Dr Anna Serlachius
Department of Psychological Medicine
The University of Auckland
PO Bag 92019
Auckland
New Zealand

Re: Peer Review of AMRF Covid-19 Project Grant, 1720008

Dear Dr Serlachius,

The Auckland Medical Research Foundation's (AMRF) 2020 Covid-19 Special Funding Round was an accelerated round held outside of the standard funding calendar, and utilised a fast-tracked application and review process that was completed within three weeks of the call for applications to ensure that the research could start immediately in response to the unprecedented need during this pandemic.

Because of the fast-tracked nature of the round, and the large response to the call for applications (42 applications submitted), the AMRF developed a brief, but robust, peer review process using New Zealand researchers. Our reviewers assessed all the applications and provided brief comments and a score out of 7, where 1 is poor and 7 is exceptional). These were used to create a shortlist of applications that were then discussed in depth via Zoom.

Dr Anna Serlachius and Dr Hiran Thabrew's application titled 'Whitu: 7 ways in 7 days – randomised controlled trial of a coping skills app to support the emotional wellbeing of young people during the COVID-19 pandemic' was favourably reviewed by the Covid-19 population health medical committee. It scored 5.04/7 (Very good) and, following a robust discussion of the top ranked grants across the fields of population health, clinical and biomedical science, was one of the seven funded applications.

Below are the brief comments that I have permission to share from four of the six reviewers of this application:

Reviewer 1: It is great to see a strong focus on Māori and Pacific participants to address inequities and the inclusion of Eva Morunga will help. However, there is no mention of Māori research approaches or methodologies being woven into the study. I would think there would be some consideration of Māori research approaches and Māori principles applied to the design of the App?

Reviewer 2: Use of technology to gauge Mental illness among youth. Strong Māori and Pasifika focus with research led by Māori/Pasifika as appropriate.

Reviewer 3: This project will build on previous work by the study team to develop and evaluate via RCT an app designed to support wellbeing among youth. App development and evaluation will be informed by Maori and Pacific researchers and participants.

Reviewer 4: Well presented study from an experienced team. The rationale is strong, but the argument in favour of an Apps as a solution is not well justified. There are many apps on the market but few have demonstrated impact on improving behaviour change and other health outcomes. Apps require data that is costly for people who may need it. It is possible that this app will include distinctively different content from other wellbeing apps and be targeted towards the specific stress associated with COVID-19.

We do hope that this overview of our peer review process, and the comments and score provided, will be sufficient to demonstrate to the editor at JMIR Research Protocols that this grant has been suitably peer reviewed. I am happy to be contacted directly if they have any questions.

Yours sincerely,

Hannah Gibbons, PhD
Research Programme Manager

100% OF YOUR DONATION DIRECTLY SUPPORTS Medical Research

Auckland Medical Research Foundation
est. 1955

P +64 9 923 1706
E hannah.gibbons@medicalresearch.org.nz
FB facebook.com/AMRFnz
T @MedResearchNZ

www.medicalresearch.org.nz
